# Prevalence of chromosome anomalies in a deer farm with fertility decline in Malaysia

**DOI:** 10.2144/fsoa-2020-0037

**Published:** 2020-06-02

**Authors:** Muhammad Sanusi Yahaya, Mohd Shahrom Salisi, Nur Mahiza Md Isa, Goh Yong Meng, Abdwahid Haron

**Affiliations:** 1 Department of Clinical Studies, Faculty of Veterinary Medicine Universiti Putra Malaysia, Serdang, Selangor 43400, Malaysia; 2 Department of Pathology and Microbiology, Faculty of Veterinary Medicine Universiti Putra Malaysia, Serdang, Selangor 43400, Malaysia; 3 Department of Theriogenology & Animal Production, Faculty of Veterinary Medicine, Usmanu Danfodiyo University, Sokoto, Nigeria; 4 Department of Preclinical Science, Faculty of Veterinary Medicine Universiti Putra Malaysia, Serdang, Selangor 43400, Malaysia

**Keywords:** chromosomal aberrations, cytogenetics, deer, fertility decline, karyotyping

## Abstract

**Background:**

A number of factors are known to reduce fertility rate in animals and one of the important categories of such factors is chromosome anomalies. They can occur with or without causing phenotypic abnormalities on animals; in some cases, they may directly affect meiosis, gametogenesis and the viability of conceptus. In many instances, balanced structural rearrangements can be transmitted to offspring, affecting fertility in subsequent generations.

**Aim:**

This work investigated the occurrence of chromosome aberrations in *Rusa timorensis*, *Rusa unicolor* and *Axis axis* raised in a nucleus deer farm in Malaysia with a history of declining fertility of unknown origin.

**Materials & methods:**

Blood samples were collected from 60 animals through venipuncture, cultured for 72 h and arrested at metaphase. SmartType^®^ and Ideokar^®^ software were used to karyotype the chromosomes.

**Results:**

We found 15 out of the 60 animals screened from both sexes harbor some form of chromosome aberration. Chromosomal aberrations exist at the rate of 25% and may not be unconnected with the observed reduced fertility on the farm. Further investigations should be carried out, especially on the offspring of the studied animals to transmission of these aberrations. The animals that are confirmed to transmit the chromosomal aberrations should be culled to arrest the propagation of their abnormalities.

## Lay abstract

Some genetic problems affect fecundity in animals; they affect reproduction thereby reducing farm output. There are reports regarding these types of problems, which links them to causing enormous economic losses in different types of animal farms. In this work, we collected blood samples from a deer farm where different breeds are kept together and fertility decline has been reported previously to check if these types of genetic problems are present on the farm. Our investigation revealed that 25% of the animals investigated, both males and female, carry a type of genetic problem that was previously reported to be associated with fertility issues. We recommend a more extensive investigation to get to the bottom of the problem, to proffer appropriate solution and to avoid economic losses on the farm.

Animal selection is an important aspect of any breeding or conservation programmes, it allows the incorporation of animals that are considered fit based on the objectives of the breeding programmes. Venison, produced from deer, is an important meat type that has the capacity to augment other regular types of meat. With the expansion of the global population, there is a growing need for efficient large-scale breeding. Fertility is an important factor, which plays a major role on the economics of livestock production. Chromosome aberrations have been implicated as a risk factor in embryo malformations and early embryo mortality in the domestic animals. The association between chromosomal aberrations and reduced fertility and/or infertility has been documented in both domestic and wild animals [1]. They can occur as numerical errors or structural rearrangements, usually with or without causing phenotypic abnormalities on carrier animals [2]. In other cases, they may directly affect meiosis, gametogenesis and the viability of conceptus [3].

In many instances, balanced structural rearrangements can be transmitted to offspring, affecting fertility in subsequent generations [2]. One of the important initial screening tests is the chromosomal aberration test [4], it excludes animals found to carry numerical or morphological defects in their chromosomes. This test has been incorporated into breeding programmes and is reported to be an effective selection criterion [5]. Chromosomal polymorphisms have been reported in many breeds of the deer, for example, using R-banding and fluorescent *in situ* hybridization, Bonnet-Garnier *et al.* have characterized chromosomal polymorphisms in several deer species due to Robertsonian translocations [6]. On the other hand, Hathaipat *et al.* have characterized the chromosomes of *Axis axis* using G-banding [7].Valeri *et al.* [8] and Cursino *et al.* [9] reported four to six karyomorphs, which resulted from intrapopulation chromosomal polymorphisms in genus Mazama. Cursino *et al.* further suggested the possibility of decline in fertility and speciation as a result of this phenomenon [9]. In another study, Salviano *et al.* [10] has reported the possibility of reproductive isolation due to intraspecific chromosome polymorphisms in the same genus, while Abril and Duarte [11] also characterized the Mazama genus using G-banded karyotypes. Huang *et al.* [12] applied comparative chromosome banding, chromosome painting and bacterial artificial chromosome (BAC) mapping to characterize the genus, while others used mitochondrial DNA to infer chromosomal polymorphisms in the same genus [13]. Although there is a growing concern of infertility in the deer in Malaysia, there is no systematic screening for chromosome aberrations, which may be associated with the observed fertility decline. Therefore, this study aimed to investigate the occurrence of chromosome aberrations in a Malaysian nucleus deer farm, which had not previously been screened.

## Materials & methods

### Sample collection & culture initiation

A total of 60 animals were sampled using heparinized vacutainer tubes through venipuncture, from the jugular vein. Within 6 h after collection, 5 ml of blood from each animal was layered onto 5 ml of Ficoll-Paque Plus^®^, 1.077 g/ml (Amersham Biosciences, Buckinghamshire, UK) in a 15 ml centrifuge tube. The samples were centrifuged for 30 min at 1000 × *g*. The peripheral blood mononuclear cell rich layer was transferred into a sterile tube, 10 ml of sterile phosphate-buffered saline (PBS) was added and the cells were re-suspended and centrifuged for 15 min at 400 × *g*. The harvested cells were finally resuspended in 3 ml of PBS [14].

Cells were cultured according to the protocols developed by Yahaya *et al.* [15]. Five drops of cell suspension were added into a culture flask containing r.p.m.I 1640 medium (Thermo Fisher Scientific, SC, USA), 10% fetal bovine serum (Sigma-Aldrich, MO, USA), mitogen (Pokeweed^®^, Sigma-Aldrich) and antibiotics (PeStrep^®^, Kepro, Maagdenburgstraat, Deventer, Netherlands). The cultures were incubated at 37°C and 5% CO_2_ for 72 h. Before termination, Colcemid was introduced to arrest the cells at metaphase. After the 72-h duration, the culture was terminated and the suspension was centrifuged for 5 min at 1800 r.p.m. The supernatant was discarded and the sediment was re-suspended in 6 ml 0.075 M KCL.

After treatment in KCL, the cells were washed in three changes of Carnoy’s fixative (a mixture of glacial acetic acid and methanol, 1:3). They were centrifuged at 1800 r.p.m. for 8 min after each wash. They were finally resuspended in 3 ml of Carnoy’s fixative and stored at 4°C for 30 min before slides were prepared.

### Metaphase preparation, chromosome banding & karyotype construction

A total of 50 μl of cell suspension was dropped onto a clean, grease-free, prechilled microscope slide from a distance of approximately 15 cm; the slides were fixed over steam for 30 s and aged for 3–5 days.

### G-banding

After aging for 3 days, the slides were rinsed in distilled water, air-dried and G-banded according the procedure described by Seabright [16] with some modifications. The slides were incubated in 0.25% freshly prepared trypsin for 15–25 s, washed in three baths of PBS to block the action of trypsin and then stained with 5 and 10% Giemsa for 5–10 minutes. The slides were viewed under light microscope at ×100 and metaphases were captured for karyotyping. This procedure was replicated for all animals.

### Karyotyping

Karyotypes were constructed using SmartType^®^ software v3.3.1 (Digital Scientific UK Ltd, England, UK) and Ideokar^®^ v1.2 (IdeoKar, Ghader Mirzaghaderi, University of Kurdistan, Sanandaj, Iran). The parameters obtained from the normal karyotypes with Ideokar were used to identify the type and nature of the chromosomal abnormality encountered (Tables 1–3). The chromosomes were designated as metacentric, acrocentric or telocentric based on the biometric method used (while methods such as fluorescent *in situ* hybridizations use genes and other DNA sequences to classify chromosomes, biometric methods use sizes and landmarks to classify them). The method uses centromeric index to classify the chromosomes; chromosome with a centromeric index of 46–50 = metacentric, 31–45 = submetacentric, 15–30 = acrocentric, <15 = telocentric 10 and less is automatically classified as telocentric [17–19]. Where;

$$CI=\frac{\text{length}\;\text{of}\;\text{p}-\text{arm}}{\left(\text{length}\;\text{of}\;\text{the}\;\text{whole}\;\text{chromosome}\right)}\times 100$$

Note: The smartType software uses chromosomes bands and lengths to construct karyotypes. The colors are used by the software to pair the chromosomes. In case there is a mistake with automatic assignment of chromosomes to their position by the software, they can be manually reassigned.

**Table 1.** Mean length of short arm chromosome, long arm chromosome, total chromosome length and centromeric index from ten metaphases of normal *Axis axis* deer in PTH, Lenggong, 2n = 66.

Chromosome	p-value	Q	TL	CI	Type
1.	2.409	5.450	7.859	30.65275	Submetacentric
2.	3.303	4.354	7.657	43.137	Submetacentric
3.	0.000	7.735	7.735	0	Telocentric
4.	0.000	4.311	4.311	0	Telocentric
5.	0.000	4.255	4.255	0	Telocentric
6.	0.000	4.205	4.205	0	Telocentric
7.	0.000	4.109	4.109	0	Telocentric
8.	0.000	3.801	3.801	0	Telocentric
9.	0.000	3.733	3.733	0	Telocentric
10.	0.000	3.704	3.704	0	Telocentric
11.	0.000	3.675	3.675	0	Telocentric
12.	0.000	3.664	3.664	0	Telocentric
13.	0.000	3.582	3.582	0	Telocentric
14.	0.000	3.520	3.52	0	Telocentric
15.	0.000	3.330	3.33	0	Telocentric
16.	0.000	3.256	3.256	0	Telocentric
17.	0.000	3.215	3.215	0	Telocentric
18.	0.000	3.204	3.204	0	Telocentric
19.	0.000	3.075	3.075	0	Telocentric
20.	0.000	3.011	3.011	0	Telocentric
21.	0.000	2.978	2.978	0	Telocentric
22.	0.000	2.903	2.903	0	Telocentric
23.	0.000	2.889	2.889	0	Telocentric
24.	0.000	2.850	2.85	0	Telocentric
25.	0.000	2.843	2.843	0	Telocentric
26.	0.000	2.813	2.813	0	Telocentric
27.	0.000	2.789	2.789	0	Telocentric
28.	0.000	2.742	2.742	0	Telocentric
29.	0.000	2.701	2.701	0	Telocentric
30.	0.000	2.645	2.645	0	Telocentric
31.	0.000	2.499	2.499	0	Telocentric
32.	0.000	2.386	2.386	0	Telocentric
X	0.000	6.889	6.889	0	Telocentric
Y	0.000	2.295	2.295	0	Telocentric

CI: Centromeric index; P: Short arm chromosome; PTH: Pusat Ternakan Haiwan; Q: Long arm chromosome; TL: Total chromosome length.

**Table 2.** Mean length of short arm chromosome, long arm chromosome, total chromosome length and centromeric index from ten metaphases of normal *Rusa timorensis* deer in PTH, Lenggong 2n = 60.

Chromosome	p-value	Q	TL	CI	Type
1.	3.134	4.944	8.077	38.80154	Submetacentric
2.	2.576	3.899	6.476	39.77764	Submetacentric
3.	2.855	3.203	6.058	47.12776	Metacentric
4.	2.158	2.506	4.665	46.25938	Metacentric
5.	1.810	2.019	3.829	47.27083	Metacentric
6.	0	5.431	5.431	0	Telocentric
7.	0	4.526	4.526	0	Telocentric
8.	0	4.038	4.038	0	Telocentric
9.	0	3.690	3.690	0	Telocentric
10.	0	3.969	3.969	0	Telocentric
11.	0	3.133	3.133	0	Telocentric
12.	0	3.133	3.133	0	Telocentric
13.	0	3.063	3.063	0	Telocentric
14.	0	2.715	2.715	0	Telocentric
15.	0	2.646	2.646	0	Telocentric
16.	0	2.924	2.924	0	Telocentric
17.	0	2.437	2.437	0	Telocentric
18.	0	2.715	2.715	0	Telocentric
19.	0	2.715	2.715	0	Telocentric
20.	0	2.924	2.924	0	Telocentric
21.	0	2.437	2.437	0	Telocentric
22.	0	2.855	2.855	0	Telocentric
23.	0	2.228	2.228	0	Telocentric
24.	0	2.297	2.297	0	Telocentric
25.	0	2.019	2.019	0	Telocentric
26.	0	2.506	2.506	0	Telocentric
27.	0	2.089	2.089	0	Telocentric
28.	0	2.367	2.367	0	Telocentric
29.	0	2.228	2.228	0	Telocentric
X	0	6.406	6.406	0	Telocentric
Y	0	2.009	2.009	0	Telocentric

CI: Centromeric index; P: Short arm chromosome; Q: Long arm chromosome; TL: Total chromosome length.

**Table 3.** Mean length of short arm chromosome, long arm chromosome, total chromosome length and centromeric index from ten metaphases of normal *Rusa unicolor* deer in PTH, Lenggong 2n = 62.

Chromosome	p-value	Q	TL	CI	Type
1.	3.234	4.544	7.778	41.57881	Submetacentric
2.	2.676	3.798	6.474	41.33457	Submetacentric
3.	2.555	3.203	5.758	44.37305	Submetacentric
4.	2.158	2.906	5.064	42.61453	Submetacentric
5.	0	5.618	5.618	0	Telocentric
6.	0	5.331	5.331	0	Telocentric
7.	0	4.527	4.527	0	Telocentric
8.	0	4.348	4.348	0	Telocentric
9.	0	4.092	4.092	0	Telocentric
10.	0	3.989	3.989	0	Telocentric
11.	0	3.743	3.743	0	Telocentric
12.	0	3.522	3.522	0	Telocentric
13.	0	3.356	3.356	0	Telocentric
14.	0	3.003	3.003	0	Telocentric
15.	0	2.904	2.904	0	Telocentric
16.	0	2.807	2.807	0	Telocentric
17.	0	2.711	2.711	0	Telocentric
18.	0	2.599	2.599	0	Telocentric
19.	0	2.402	2.402	0	Telocentric
20.	0	2.387	2.387	0	Telocentric
21.	0	2.304	2.304	0	Telocentric
22.	0	2.287	2.287	0	Telocentric
23.	0	2.258	2.258	0	Telocentric
24.	0	2.201	2.201	0	Telocentric
25.	0	2.177	2.177	0	Telocentric
26.	0	2.096	2.096	0	Telocentric
27.	0	2.069	2.069	0	Telocentric
28.	0	2.007	2.007	0	Telocentric
29.	0	1.899	1.899	0	Telocentric
30.	0	1.802	1.802	0	Telocentric
X	0	6.518	6.518	0	Telocentric
Y	0	2.038	2.038	0	Telocentric

CI: Centromeric index; P: Short arm chromosome; Q: Long arm chromosome; TL: Total chromosome length.

## Results

To assess the chromosome anomalies discovered in this work, a comprehensive analysis of the karyotype from the apparently normal animals was performed using the appropriate software (Tables 1–3). These were later used as the standard for assessing the discovered anomalies to identify the chromosomes involved in translocation. The mean lengths of short and long arm chromosome, the total chromosome length and the centromeric indices, were used to characterize the chromosomes. *A. axis* had two submetacentric pairs, 30 pairs of telocentric autosomes and two pairs of telocentric sex chromosomes. In *Rusa timorensis* there were 29 pairs of autosomes, three pairs of submetacentric, two pairs of metacentric and the sex chromosomes in this breed were telocentric. *Rusa unicolor*, on the other hand, had four pairs of submetacentric, 26 pairs of telocentric autosomes and telocentric X- and Y-chromosomes. Chromosomes are classified as metacentric, submetacentric and telocentric, because the acrocentric and telocentric chromosomes fall within the same group based on their centromeric indices. This is consistent with the literature, while Bonnet-Garnier *et al.* [6] classified the nonmetacentric and nonsubmetercentric chromosomes as acrocentric, Hathaipat *et al.* [7], classified them as telocentric. However, this does not change a species chromosomes’ number or the fundamental number chromosomes, for example, the karyotype of *A. axis* has not changed from 2n = 66 and fundamental number = 70 in both cases. Therefore, classifying the chromosomes as acrocentric or metacentric does not constitute a problem in cytogenetics.

Pooling the entire population, 25% of the animals screened were demonstrated to carry chromosomal anomalies. In *R. timorensis*, 25 individuals were screened and 24% of them were found to carry chromosomal anomalies. In *R. unicolor*, 25% of the 20 individuals screened, carry chromosomal anomalies while 28% of *A. axis* were found to be abnormal (Table 4).

**Table 4.** Total number of animals characterized and the proportion of normal to those with chromosomal aberrations.

Animals	Sex	Number of animals	Normal karyotype	Abnormal karyotype
*Rusa timorensis*	Male	10	7 (70%)	3 (30%)
	Female	15	12 (80)	3 (20%)
*Rusa unicolor*	Male	7	5 (71%)	2 (29%)
	Female	13	10 (77)	3 (23)
*Axis axis*	Male	4	2 (50%)	2 (50%)
	Female	11	9 (81%)	2 (19%)
Total		60	45 (75%)	15 (25%)

**Figure 1.**
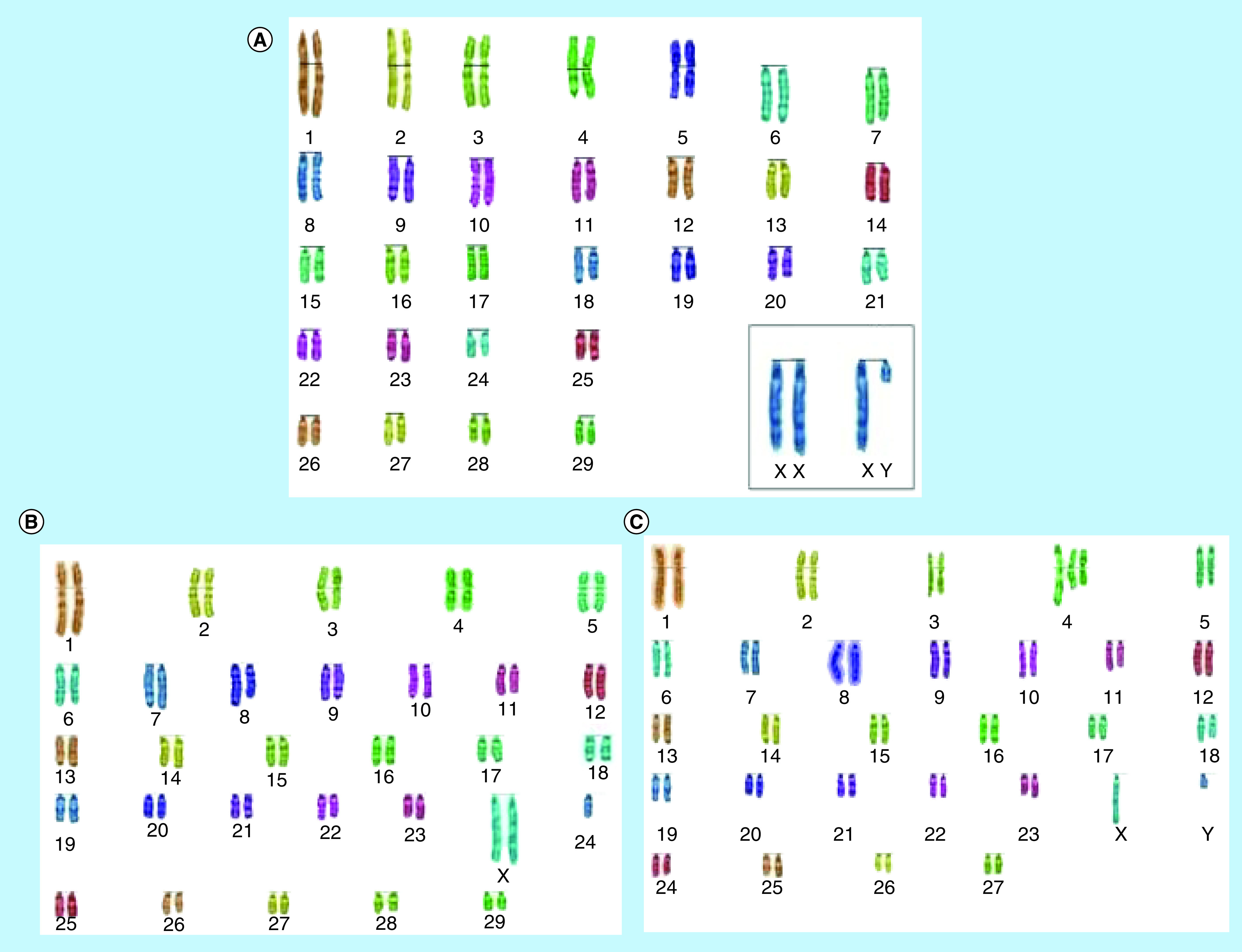
Karyograms of *Rusa timorensis*. **(A)** Normal karyotype of *Rusa timorensis*, **(B)** abnormal karyotype in a deer hind (2n = 59 instead of 60, likely rb [8,24]), **(C)** abnormal karyotype in a deer buck (2n = 57 instead of 60) using the SmartType^®^ software. rb: Robertsonian.

**Figure 2.**
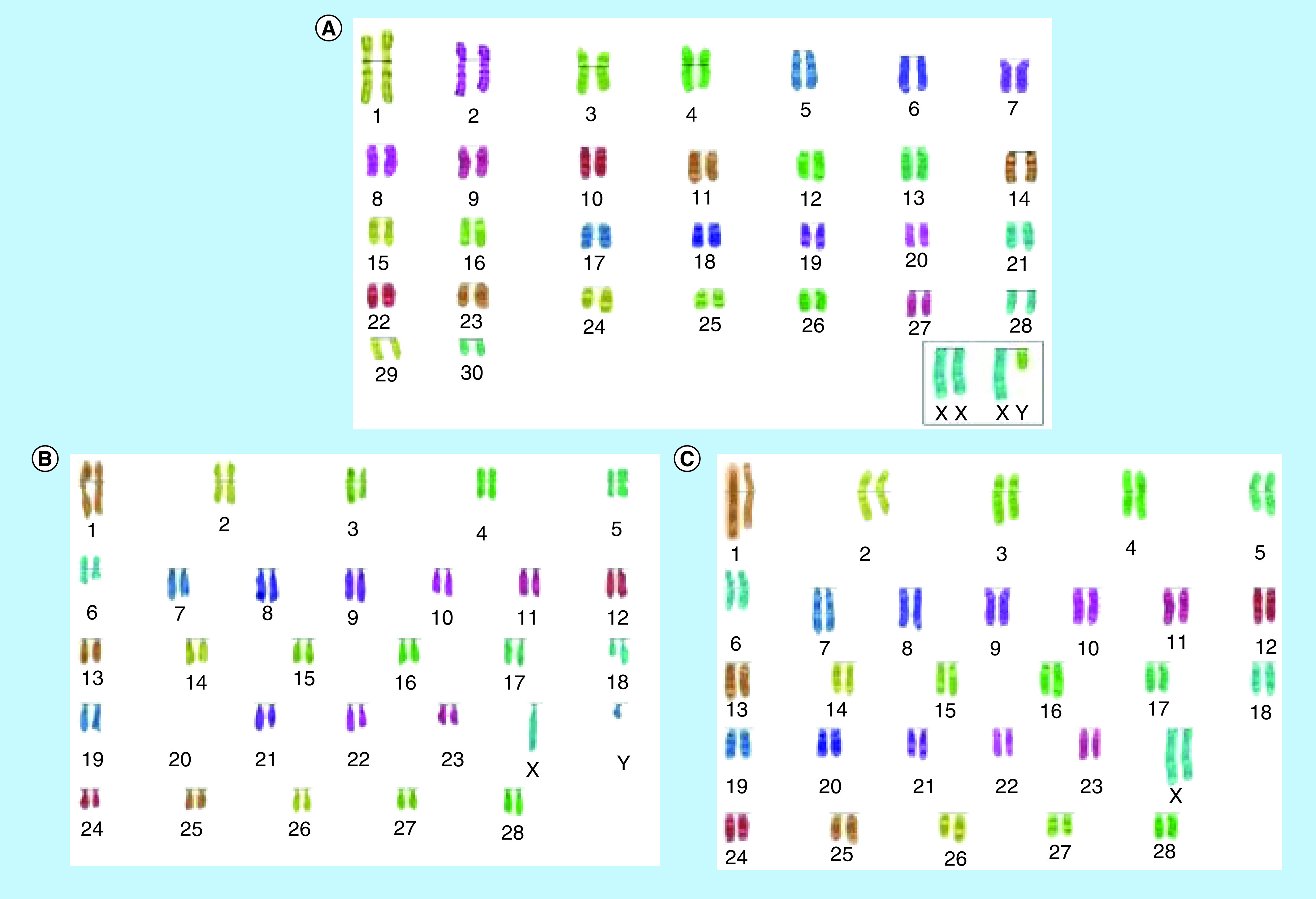
Karyograms of *Rusa unicolor*. **(A)** Normal karyotype of *Rusa unicolor*, **(B)** abnormal karyotype in a deer buck (2n = 58 instead of 62), **(C)** abnormal karyotype in a deer hind (2n = 58 instead of 62) using the SmartType^®^ software.

**Figure 3.**
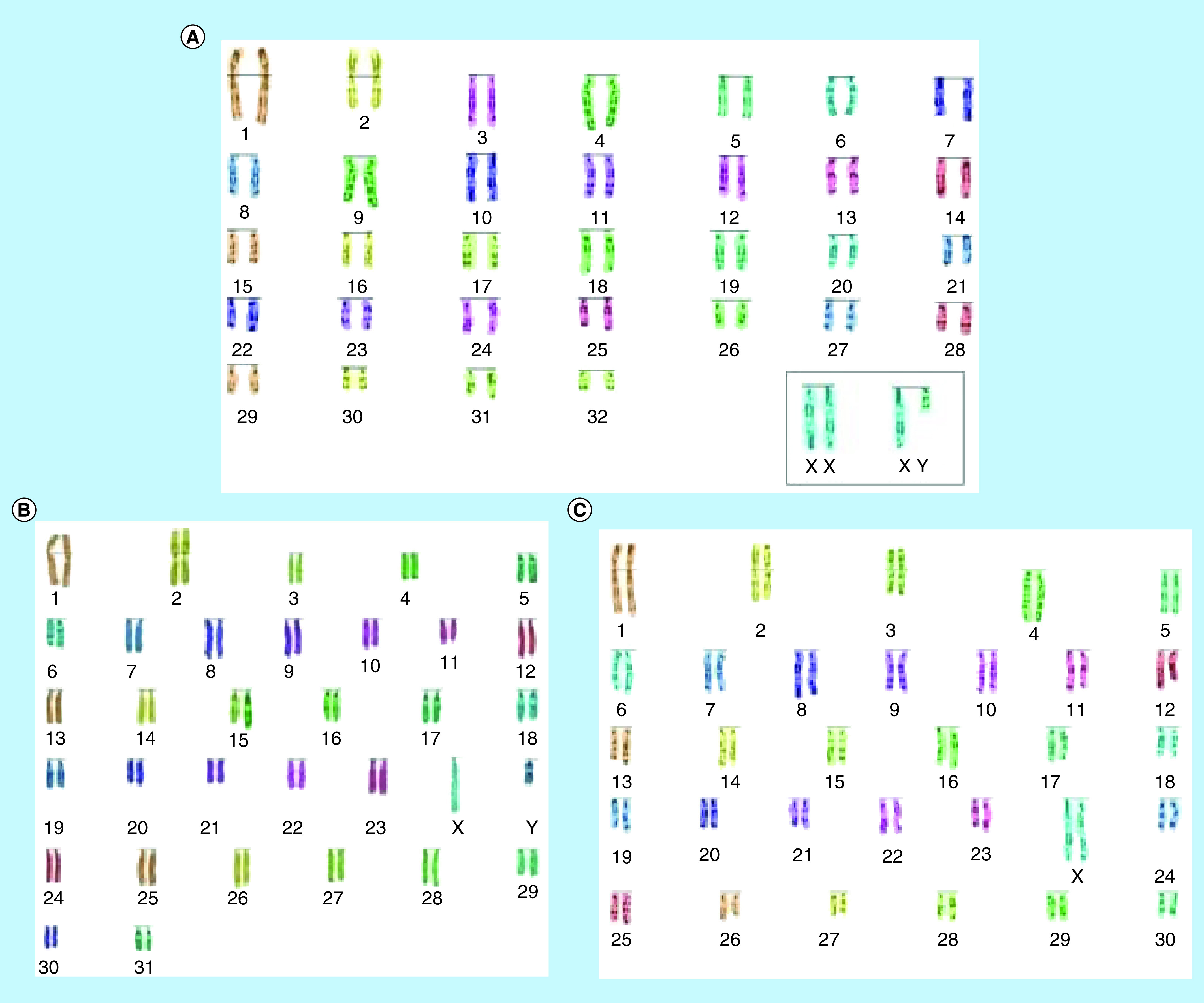
Karyograms of *Axis axis*. **(A)** Normal karyotype of *Axis axis*, **(B)** abnormal karyotype in a deer buck (2n = 64 instead of 66), **(C)** abnormal karyotype in a deer hind (2n = 62 instead of 66) using the SmartType^®^ software.

The Figures 1–3 highlight the most frequent set of abnormal karyotypes from the three breeds of deer in this work. In *R. timorensis* the most frequent abnormal female karyotype composed of 57 instead of the normal 60 chromosomes. Additional metacentric chromosome was observed in this set, which appear to result from Robertsonian fusion between chromosome 8 and 24 rob (8, 24) (Figure 1). In the male, however, there was translocation between unidentified chromosomes and an apparent Robertsonian translocation, which may be, associated with chromosome 1 and 6, considering the length of the chromosomes. In *R. unicolor* the abnormal male and female were both observed to carry 58 chromosomes instead of 62, however, they carry a pair of additional submetacentric chromosomes, making the number of metacentric chromosomes six pairs rather the than five (Figure 2). In *A. axis*, there were 62 chromosomes in the males and 64 in the females instead of the 66. Three pairs of metacentric chromosomes were found in the females while two pairs were found in males (Figure 3). All anomalies discovered resulted from translocation between chromosomes, possibly because of prolonged interbreeding.

## Discussion

Structural chromosome abnormalities are known to affect fertility rate in domestic animals. Checking these abnormalities is an important mechanism used to exclude from breeding programs to prevent the transmission of the undesirable traits contained in their genome [20]. Improving fertility traits such as litter size, entails mitigating the effects of embryonic loss during pregnancy. Chromosome aberrations have been known to increase the risk of embryonic malformations and early mortality in livestock [21]. To checkmate the negative impact of chromosome anomalies, a number of countries have developed cytogenetic screening programs, where animals intended for breeding are tested for chromosomes aberration and appropriate measures are instituted to prevent the economic losses associated with them. Such countries include France and Canada, both in the pork industry [22,23]. Breeding boars and sows are screened for chromosomal anomalies, with animals found with anomalies being culled from the programs. This resulted in the most precise estimate of the occurrence of chromosomal anomalies in livestock and in improving economic gains when animals with chromosomes aberrations are culled.

The global meat industry is huge, with an estimated net value of US$945.7 billion in 2018 [24]. It is therefore, imperative to protect the industry from the negative effects of genetic disorders. In this study, the normal karyotypes (Tables 1–3) are similar to earlier reports [7] in *A. axis* [25], in *R. timorensis* and [26] in *R. unicolor*. These were instrumental in revealing the specific anomalies and the specific chromosomes in which they occur in the study population. The high prevalence of chromosome anomalies in the study underscores the importance of cytogenetic screening in breeding farms. It also suggests a serious underlying problem, which must be addressed as quickly as possible within the population. The occurrence of chromosomal polymorphisms identified in this study points to an ongoing process, which may not be unconnected with observed fertility decline in this deer population. However, a larger sample and more robust techniques such as the fluorescent *in situ* hybridization should be conducted to further elucidate the mechanisms involved in this process.

## Future perspective

Breeding soundness evaluation methods ensure the continued improvement of animal production; they ensure the exclusion of unfit animals from breeding programs. One of the most important of these methods is the cytogenetic evaluation of animals intended for breeding. The combination of both conventional and molecular cytogenetics, which offers a finer resolution for genetic disease detection are gradually becoming more prominent in animal breeding farms. The advancement and availability of molecular cytogenetic techniques will make the integration of these methods into the mainstream of the animal industry in the near future.

**Summary points**

- One of the important group of factors associated with fertility decline in animals is chromosome aberration.
- They may or may not cause phenotypic abnormalities on animals.
- Sometimes they directly affect meiosis, gametogenesis and the viability of conceptus.
- Balanced structural rearrangements often are transmitted to offspring, affecting fertility in subsequent generations.
- This work investigated the occurrence of chromosome aberrations in *Rusa timorensis, Rusa unicolor* and *Axis axis* raised in a nucleus deer farm in Malaysia with a history of declining fertility of unknown origin.
- Blood samples were collected from 60 animals through venipuncture, cultured for 72 h and arrested at metaphase.
- Light microscope at ×100 was used to capture the metaphase spreads.
- SmartType^®^ and Ideokar^®^ software were used to karyotype the chromosomes.
- A total of 15 out of the 60 animals screened from both sexes were found to harbor some form of chromosome aberration.
- These aberrations exist at the rate of 25% in the study population and may not be unconnected with the observed reduced fertility on the farm.
- Further investigations should be carried out, especially on the offspring of the studied animals to check for possible transmission of these aberrations.
- Both parents and offspring that harbor chromosomal aberrations should be culled to arrest the propagation of their abnormalities within the farm.
